# Genome Sequences of Three Novel Isolates of Human Parainfluenza Virus 2 Associated with Acute Respiratory Infection

**DOI:** 10.1128/genomeA.00784-17

**Published:** 2017-08-17

**Authors:** J. L. Kennedy, J. C. Kincaid, K. C. Schwalm, A. N. Stoner, T. J. Abramo, T. M. Thompson, O. Hardin, C. Putt, D. L. Dinwiddie

**Affiliations:** a Department of Pediatrics, Arkansas Children’s Research Institute, University of Arkansas for Medical Sciences, Little Rock, Arkansas, USA; b Department of Internal Medicine, University of Arkansas for Medical Sciences, Little Rock, Arkansas, USA; c Department of Pediatrics, University of New Mexico Health Sciences Center, Albuquerque, New Mexico, USA; d Clinical Translational Sciences Center, University of New Mexico Health Sciences Center, Albuquerque, New Mexico, USA

## Abstract

Using target capture of viral nucleic acid and next-generation sequencing, we generated the genome sequences of three novel human parainfluenza virus 2 isolates. Isolates ACRI_0185 (GenBank accession number MF077311), ACRI_0230 (MF077312), and ACRI_0248 (MF077313) were collected in October 2016, February 2017, and March 2017, respectively, from pediatric patients with acute respiratory infection in Arkansas.

## GENOME ANNOUNCEMENT

Human parainfluenza viruses (hPIVs) are a major cause of acute respiratory infection (ARI) in children; collectively, they are second only to respiratory syncytial viruses as causes of hospitalization (1–3). Each of the four hPIVs has been shown to manifest as both upper and lower respiratory tract disease (4). Though not as common as hPIV1, hPIV2 is an established cause of croup (4). The genome of hPIV2 comprises ∼15,650 nucleotides (nt) (5).

Here, we present three novel genome sequences of hPIV2 isolates from patients who presented with cold symptoms to the emergency department (ED) at the Arkansas Children’s Hospital in Little Rock, Arkansas, USA. Patient ACRI_0185 was a 15-year-old African American male with severe persistent asthma, seen October 2016 with worsening asthma symptoms over the previous 10 days. In the ED, the patient had normal vital signs with an SpO_2_ of 96% on room air. He required three doses of nebulized albuterol/ipratropium and oral steroids to improve wheezing, and he was discharged home with a steroid burst. Patient ACRI_0230 was a 5-year-old healthy Caucasian male who presented in February 2017 with persistent cough for 2 weeks. He was afebrile with normal vital signs. Auscultation of the chest revealed bilaterally equal coarse breath sounds without wheezing. The patient was discharged home with supportive measures. Patient ACRI_0248 was a 4-year-old healthy Caucasian male who was seen in March 2017 with a 2-day history of barky cough. He was afebrile with normal vital signs and an SpO_2_ of 97%. He was diagnosed with croup and given dexamethasone intramuscularly. He was discharged home with continued supportive measures.

Nasopharyngeal swabs were collected after consent for participation in an ongoing study approved by the institutional review board. An Illumina stranded-RNA library was created from isolated RNA, and hybridization-based enrichment was performed using the University of New Mexico’s ResVir respiratory viral panel probe set, which contains 5,683 hybridization probes designed to be complementary to coding sequence regions of 24 human respiratory viruses. Next-generation sequencing was performed on an Illumina MiSeq platform using V3 chemistry and paired 75-bp reads.

Sample ACRI_0185 had 13,757 sequencing reads align to the hPIV2 RefSeq genome (NC_003443), which resulted in a mean coverage of 65×. Samples ACRI_0230 and ACRI_0238 had 94,858 sequencing reads with a 274× mean coverage and 93,696 sequencing reads with a 217× mean coverage, respectively. Alignment-guided assembly was used to generate isolate genome sequences (CLC Genomics Workbench version 9), which were annotated using the ViPR Genome Annotator (6). In comparison to NC_003443, isolates ACRI_0185, ACRI_0239, and ACRI_0248 showed significant variability with 616, 681, and 696 nt differences, of which 97, 104, and 108 were predicted to cause amino acid changes. In comparison with each other at the genome level, isolates ACRI_0230 and ACRI_0248 had 185 nt differences, whereas ACRI_0185 had more than 440 nt differences to each of the other two isolates. Phylogenetic analysis through nearest-neighbor joining revealed that all three isolates grouped in a genotype with hPIV2 strain V94 (AF533010) and samples collected in Washington state, USA, represented by samples KY674947 and KY674948, which were distinct from the genotype represented by samples KY674949 to KY674952.

### Accession number(s).

The whole-genome sequences of isolates ACRI_0185, ACRI_0230, and ACRI_0248 have been deposited in GenBank under the accession numbers MF077311, MF077312, and MF077313, respectively.

## References

[1] Berman S 1991 Epidemiology of acute respiratory infections in children of developing countries. Rev Infect Dis 13(suppl 6):S454–S462. doi:10.1093/clinids/13.Supplement_6.S454. 1862276

[2] Fowlkes A, Giorgi A, Erdman D, Temte J, Goodin K, Di Lonardo S, Sun Y, Martin K, Feist M, Linz R, Boulton R, Bancroft E, McHugh L, Lojo J, Filbert K, Finelli L, IISP Working Group 2014 Viruses associated with acute respiratory infections and influenza-like illness among outpatients from the Influenza Incidence Surveillance Project, 2010–2011. J Infect Dis 209:1715–1725. doi:10.1093/infdis/jit806. 24338352 PMC5749912

[3] Glezen WP, Frank AL, Taber LH, Kasel JA 1984 Parainfluenza virus type 3: seasonality and risk of infection and reinfection in young children. J Infect Dis 150:851–857. doi:10.1093/infdis/150.6.851. 6094674

[4] Henrickson KJ 2003 Parainfluenza viruses. Clin Microbiol Rev 16:242–264. doi:10.1128/CMR.16.2.242-264.2003. 12692097 PMC153148

[5] Skiadopoulos MH, Vogel L, Riggs JM, Surman SR, Collins PL, Murphy BR 2003 The genome length of human parainfluenza virus type 2 follows the rule of six, and recombinant viruses recovered from non-polyhexameric-length antigenomic cDNAs contain a biased distribution of correcting mutations. J Virol 77:270–279. 12477832 10.1128/JVI.77.1.270-279.2003 PMC140631

[6] Pickett BE, Sadat EL, Zhang Y, Noronha JM, Squires RB, Hunt V, Liu M, Kumar S, Zaremba S, Gu Z, Zhou L, Larson CN, Dietrich J, Klem EB, Scheuermann RH 2012 ViPR: an open bioinformatics database and analysis resource for virology research. Nucleic Acids Res 40:D593–D598. doi:10.1093/nar/gkr859. 22006842 PMC3245011

