# Erratum for Kennedy et al., “Genome Sequences of Three Novel Isolates of Human Parainfluenza Virus 2 Associated with Acute Respiratory Infection”

**DOI:** 10.1128/genomeA.01145-17

**Published:** 2017-10-19

**Authors:** J. L. Kennedy, J. C. Kincaid, K. C. Schwalm, A. N. Stoner, T. J. Abramo, T. M. Thompson, O. Hardin, C. Putt, D. L. Dinwiddie

**Affiliations:** a Department of Pediatrics, Arkansas Children’s Research Institute, University of Arkansas for Medical Sciences, Little Rock, Arkansas, USA; b Department of Internal Medicine, University of Arkansas for Medical Sciences, Little Rock, Arkansas, USA; c Department of Pediatrics, University of New Mexico Health Sciences Center, Albuquerque, New Mexico, USA; d Clinical and Translational Science Center, University of New Mexico Health Sciences Center, Albuquerque, New Mexico, USA

## ERRATUM

Volume 5, no. 33, e00784-17, 2017, https://doi.org/10.1128/genomeA.00784-17. Page 1: The affiliation line and the superscript letters associated with the article byline should read as given above.

